# Locked-in Syndrome in a Young Patient Due to SARS-CoV-2: A Case Report

**DOI:** 10.3389/fmed.2020.574690

**Published:** 2020-10-15

**Authors:** Saud Bin Abdul Sattar, Qasim Zafar Iqbal, Muhammad Adnan Haider, Zeeshan Zia, Muhammad Raphay Khan Niazi, Muhammad Hanif, Mukarram Jamat Ali, Muhammad Aslam Khan

**Affiliations:** ^1^Staten Island University Hospital, Northwell Health, New York, NY, United States; ^2^Allama Iqbal Medical College, Lahore, Pakistan; ^3^Khyber Medical College, Hayatabad Medical Complex, Peshawar, Pakistan; ^4^King Edward Medical University, Lahore, Pakistan

**Keywords:** COVID-19, SARS- CoV-2, thrombotic complication, hypercoagulability, locked-in syndrome

## Abstract

Coronavirus disease 2019 (COVID-19), apart from commonly involving the respiratory system, has its impact on the central nervous system, with a wide spectrum of clinical presentations ranging from headaches to ischemic strokes. The ongoing research regarding this novel disease has found that there is a very high prevalence of thrombotic episodes especially in critically ill patients when compared to severe presentation of other viral illnesses. This COVID-19-associated coagulopathy has a very complex etiology with the ability to form thrombus in arteries, veins, and microvasculatures of different organs. We present a unique case of a young woman with underlying COVID-19 who unfortunately developed locked-in syndrome due to bilateral pontine infarction during the course of her illness.

## Introduction

Locked-in syndrome is a state of motor paralysis involving all the voluntary muscles of four limbs along with dysarthria, however with preserved alertness and consciousness. This tragic state is rare and often caused by ischemic stroke in midbrain affecting cortico-spinal, cotico-bulbar, and cortico-pontine neuronal tracks. This is the very first case report of its kind in which the cause of ischemic stroke was this novel viral disease. Severe acute respiratory syndrome coronavirus-2019 (SARS-CoV-2019) infection that emerged in Wuhan, China in December 2019 (1) has been declared a global pandemic by World Health Organization (WHO). Where most patients with COVID-19 present with symptoms of cough, fever, fatigue, and dyspnea (2), some also have neurological manifestations like headache, anosmia, meningitis, encephalitis, Guillain–Barré syndrome, and acute cerebrovascular diseases (3). There have been many cases of ischemic stroke that have been reported in patients with confirmed COVID-19. There have been young patients without any underlying risk factors studied that developed ischemic stroke, indicating that COVID-19 has a major role in causing these ischemic strokes (4–7). Mao et al. reports that acute cerebrovascular accidents are more common in patients having a severe COVID-19 disease compared to those with less severe disease (8). Our patient uncharacteristically developed locked-in syndrome despite initially presenting with respiratory symptoms of worsening dyspnea and relentless productive cough.

## Case

A 25-year-old woman with past medical history of hypertension and diabetes mellitus type I presented to the Emergency Department (ER) with symptoms of dry cough, low-grade fever, and worsening shortness of breath for 1 week. In the emergency room, the triage vital signs showed that she was hypoxic to 70% on pulse oximetry, which improved to 96% on 6 L of supplemental oxygen via nasal cannula. Considering the COVID-19 pandemic and her typical symptoms, a nasopharyngeal swab for COVID-19 PCR was done in the ER, which eventually came out as positive.

**Figure 1:**
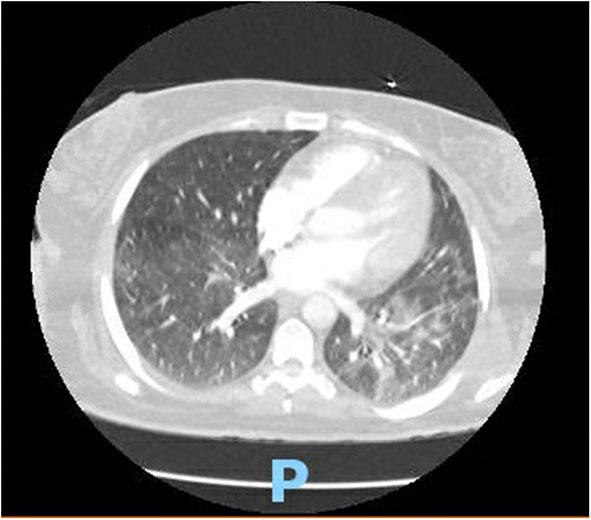
CT chest showing typical BL ground glass opacifications in lungs: a hallmark of SARS-CoV-2 infection.

On hospital day 2, she developed acute respiratory syndrome (ARDS), and she eventually had to be intubated requiring mechanical ventilation. Computed tomography of the chest showed interstitial infiltrate dictating the severity of the patient (Figure 1). Eventually, after 8 days of requiring high fraction of oxygen (FiO_2_) up to 100% and positive end expiratory pressure (PEEP) of >12, her lung compliance started to improve, and we were able to decrease her FiO_2_ and PEEP requirements. She was off sedation, and we attempted several unsuccessful spontaneous awakening and breathing trials. To evaluate her for unresponsiveness despite being off sedation, a computed tomography of the head without contrast and electroencephalography were done, which came back as unremarkable for any acute findings.

**Figure 2:**
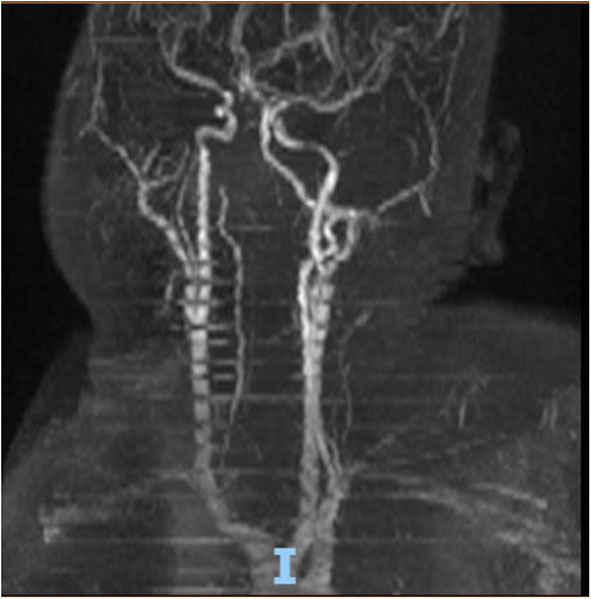
MRA neck showing patent circulations of carotid arteries.

**Figure 3:**
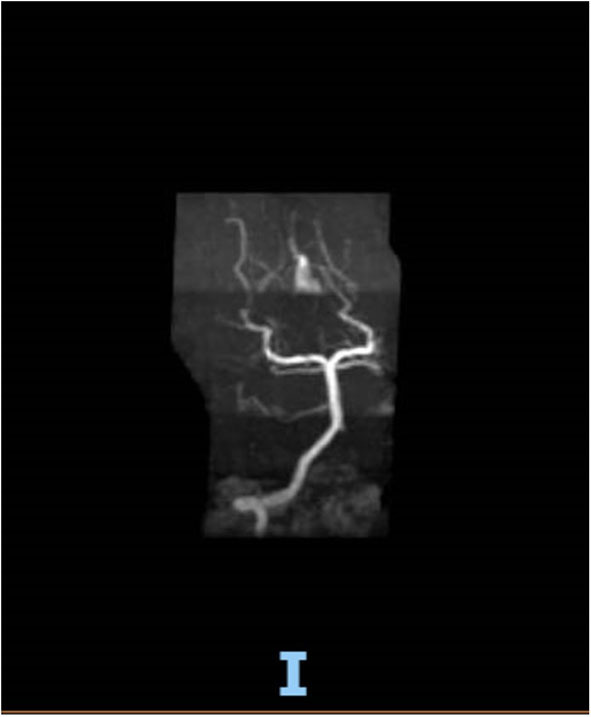
MRA brain with occluded right vertebral artery and patent basilar artery.

**Figure 4:**
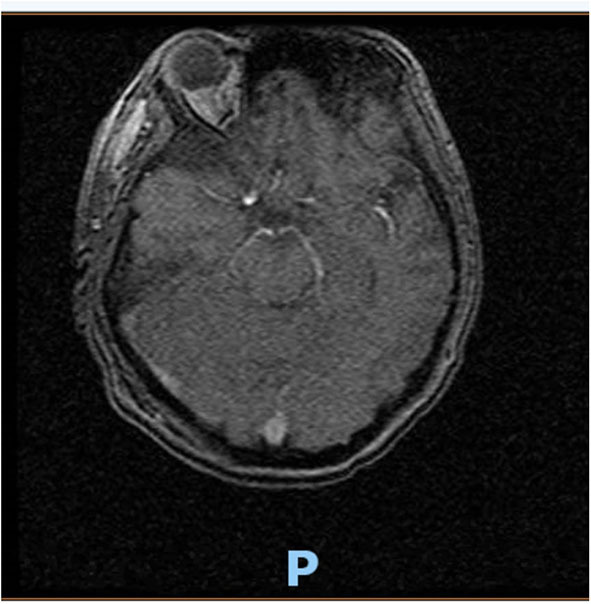
MRI brain (cross-section at pons) consistent with acute infarct of pons.

On hospital day 16, the patient finally opened her eyes and started following simple commands such as blinking of her eyelids. She was just able to respond to any command by her vertical eye ball movement and blinking of eyelids but continued to not show any movement in all four of her extremities. A repeat CT scan of the head on day 16 was also unremarkable for any acute intracranial pathology. Neurology was consulted; after 6 days off sedation on physical examination, patient was arousable to voice and tactile stimulation by opening of her eyes and was able to track objects with eye. Bilateral pupillary reflex, corneal reflex, doll's eye reflex, and gag reflex were intact. The patient's National Institutes of Health Stroke Scale (NIHSS) score was 27, showing severe stroke with total motor impairment of all extremities. A magnetic resonance imaging of the brain to rule out stroke, a magnetic resonance angiography to rule out arterial stenosis, an echocardiography to rule out cardiac source of any emboli, and a lumbar puncture for cerebrospinal fluid analysis were done. The results of the magnetic resonance angiography (MRA) of the neck (Figure 2), echocardiography, and cerebrospinal fluid (CSF) analysis was unremarkable; however, MRA of the head (Figure 3) and a non-contrast MRI of the brain (Figure 4) showed multiple foci of restricted diffusion within the pons, compatible with acute infarcts.

Patient was on deep vein thrombosis (DVT) prophylaxis Heparin 5,000 U subcutaneous every 8 h from day 1 of hospitalization. After finding acute infarct and occluded vertebral artery, full anticoagulation with intravenous Heparin 16 U/h was started with the goal of activated partial thromboplastin time (aPTT) of 60–90 s. As shown (Figure 3), there was a decreased flow in the distal right vertebral artery suspicious of blood vessel occlusion. Basilar artery is patent on the MRA (Figure 3). Given the bilateral nature of acute pontine central infarct as shown in MRI (Figure 3), the culprit basilar thrombus originated from the occluded right vertebral artery, was stuck for a time being in the basilar artery, lead to bilateral stroke, and resolved later (Figure 3). It is very less likely that a bilateral pontine infarction, leading to locked-in syndrome, can be caused by direct thromboembolic occlusion of basilar perforator arteries.

Neurologist recommended full hypercoagulable workup including platelets, protein S, protein C, antithrombin-III, PT, aPTT, d-dimer, antiphospholipid (APL) antibody isotypes, fibrinogen, and fibrin split products. Normal platelet count of around 285,000/μl and normal PT and aPTT excluded disseminated intravascular coagulation (DIC). Raised inflammatory markers, i.e., fibrinogen level, d-dimers, CRP, and ferritin are the hallmark of cytokine storm leading to sepsis-induced coagulopathy. Considering the area of ischemic stroke and the fact that there was no improvement from locked-in syndrome after 29 days, on the 30th day, a tracheostomy and percutaneous endoscopic gastrostomy was performed, and the patient was sent to a nursing home later on minimal ventilator settings.

## Discussion

We describe a case of locked-in syndrome secondary to COVID-19, leading to a tragic state of quadriplegia with sparing of consciousness and eye movements with clinical, serological, and neuroimaging evidence. The infarction of the midbrain at central pons can lead to this quadriplegic state known as locked-in syndrome (9), and this could be the very first case reported where COVID-19-associated coagulopathy led to a pseudocoma state of locked-in syndrome with acute, isolated bilateral pontine infarction. To the best of our knowledge, only one case of COVID-19-associated locked-in syndrome has been reported and that was due to acute polyradiculoneuropathy (10). The systemic infection of SARS-CoV-2 can lead to cytokine production (mainly IL-6) called “cytokine storm” as a part of innate immunity (11). The cytokine inflammatory response leads to activation of procoagulation pathways. Phosphatases (derived from viruses) activate platelets, mast cells, and factor XII (FXII), causing hypercoagulation through activation of intrinsic coagulation pathway. This also results in elevation of D-dimers, fibrinogen level, and C-reactive protein (12). The cytokines also activate endothelial cells with resultant endothelial injury, leading to microthrombi formation in vessels with subsequent ischemia and multiple organ failure (13). Another pathophysiological mechanism through which SARS-CoV-2 can cause acute cerebrovascular accidents is through endothelial injury mediated by depletion of angiotensin-converting enzyme 2 (ACE2). ACE2 receptors are expressed on lungs, intestine, and brain (14). Overexpression of ACE2 in neuronal cells or endothelial progenitor cells protects the brain from ischemic stroke (15). ACE 2 is cardio- and neuroprotective and acts by countering the effects of angiotensin converting enzyme 1 (ACE1) and angiotensin 2(AT2) in the renin–angiotensin–aldosterone system. SARS-CoV-2 binds to ACE2 receptors and depletes ACE2, leaving ACE1 unopposed with production of AT2, which worsens lung injury with proinflammatory and organ damaging effects.

The possible mechanism of COVID-19-associated coagulopathy and thrombus formation therefore can be summarized by four major mechanisms. The first is COVID-19-induced cytokine storm that activates host immune defense in order to protect the spread of the virus, leading to activation of coagulation cascade in the blood. The second is platelet activation by these proinflammatory cytokines. Third would be direct endothelial involvement causing apoptosis of endothelial cells exposing the subendothelial matrix, which acts as a potent trigger for platelet aggregation and thrombus formation. The last one could be because of fibrinolytic suppression caused by decreased activity of plasminogen activator and elevated release of plasminogen activator inhibitors. All these mechanisms are hence responsible for “sepsis induced coagulopathy,” which is related with the severity of COVID-19 (16, 17).

## Conclusion

Physicians including neurologists should be aware of the fact that COVID-19 disease can cause acute ischemic strokes. The control of COVID-19 is our biggest priority currently, but we should not neglect COVID-19-associated strokes because early provision of anticoagulation can decrease the long-term mortality and morbidity in these patients. It has now been advised that prophylactic use of anticoagulants should be given to ameliorate the potential risk of hypercoagulopathy associated with COVID-19. Some physicians are also advising to add antiplatelet drugs to prevent arterial thrombus formation, but the risk of bleeding could be a limiting factor in this practice. Further research is warranted to understand the COVID-19-associated coagulopathy and thrombus formation in arteries to prevent fatal episodes of acute coronary and cerebrovascular accidents like the one seen in our patients.

## Data Availability Statement

The original contributions presented in the study are included in the article/supplementary material, further inquiries can be directed to the corresponding author/s.

## Ethics Statement

A written, informed consent was obtained from the patient/legal representative for the publication of this case report (including all data and images).

## Author Contributions

All authors listed have made a substantial, direct and intellectual contribution to the work, and approved it for publication.

## Conflict of Interest

The authors declare that the research was conducted in the absence of any commercial or financial relationships that could be construed as a potential conflict of interest.
